# Inhibition of Glycogen Synthase Kinase-3β Prevents Remifentanil-Induced Hyperalgesia via Regulating the Expression and Function of Spinal N-Methyl-D-Aspartate Receptors In Vivo and Vitro

**DOI:** 10.1371/journal.pone.0077790

**Published:** 2013-10-16

**Authors:** Yize Li, Haiyun Wang, Keliang Xie, Chunyan Wang, Zhuo Yang, Yonghao Yu, Guolin Wang

**Affiliations:** 1 Department of Anesthesiology, Tianjin Medical University General Hospital, Tianjin, P. R. China; 2 Tianjin Research Institute of Anesthesiology, Tianjin, P. R. China; 3 Medical School, Nankai University, Tianjin, P. R. China; University of Kentucky Medical Center, United States of America

## Abstract

A large number of experimental and clinical studies have confirmed that brief remifentanil exposure can enhance pain sensitivity presenting as opioid-induced hyperalgesia (OIH). N-methyl-D-aspartate (NMDA) receptor antagonists have been reported to inhibit morphine analgesic tolerance in many studies. Recently, we found that glycogen synthase kinase-3β (GSK-3β) modulated NMDA receptor trafficking in a rat model of remifentanil-induced postoperative hyperalgesia. In the current study, it was demonstrated that GSK-3β inhibition prevented remifentanil-induced hyperalgesia via regulating the expression and function of spinal NMDA receptors in vivo and in vitro. We firstly investigated the effects of TDZD-8, a selective GSK-3β inhibitor, on thermal and mechanical hyperalgesia using a rat model of remifentanil-induced hyperalgesia. GSK-3β activity as well as NMDA receptor subunits (NR1, NR2A and NR2B) expression and trafficking in spinal cord L_4_-L_5_ segments were measured by Western blot analysis. Furthermore, the effects of GSK-3β inhibition on NMDA-induced current amplitude and frequency were studied in spinal cord slices by whole-cell patch-clamp recording. We found that remifentanil infusion at 1 μg·kg^-1^·min^-1^ and 2 μg·kg^-1^·min^-1^ caused mechanical and thermal hyperalgesia, up-regulated NMDA receptor subunits NR1 and NR2B expression in both membrane fraction and total lysate of the spinal cord dorsal horn and increased GSK-3β activity in spinal cord dorsal horn. GSK-3β inhibitor TDZD-8 significantly attenuated remifentanil-induced mechanical and thermal hyperalgesia from 2 h to 48 h after infusion, and this was associated with reversal of up-regulated NR1 and NR2B subunits in both membrane fraction and total lysate. Furthermore, remifentanil incubation increased amplitude and frequency of NMDA receptor-induced current in dorsal horn neurons, which was prevented with the application of TDZD-8. These results suggest that inhibition of GSK-3β can significantly ameliorate remifentanil-induced hyperalgesia via modulating the expression and function of NMDA receptors, which present useful insights into the mechanistic action of GSK-3β inhibitor as potential anti-hyperalgesic agents for treating OIH.

## Introduction

Opioids have been regarded as the most effective analgesics for management of acute, chronic and cancer pain[1]. Remifentanil is an ultra-short-acting μ-opioid receptor agonist. Due to its reliability, rapid onset and predictable rapid recovery profile, remifentanil has been widely used in clinical practice with little risk of delayed postoperative recovery or respiratory depression[2]. However, remifentanil-induced hyperalgesia is more rapid and frequent than other opioids[3]. Opioid-induced hyperalgesia (OIH) occurs after a brief (

< 60 min) exposure to remifentanil and contributes to an increase in postoperative pain[4-6].

The glutamatergic receptor system, especially N-methyl-D-aspartate (NMDA) receptors, plays a pivotal role in synaptic plasticity and chronic pain formation. NMDA receptors are highly permeable to Calcium (Ca^2+^), and Ca^2+^ influx through NMDA receptors is essential for synaptogenesis, experience-dependent synaptic remodeling and long-lasting changes in synaptic efficacy such as long-term potentiation (LTP) and long-lasting depression (LTD)[7,8]. NMDA receptors are heteromeric proteins composed of three subunits, NR1, NR2A-D and NR3[9,10]. The NR1 subunit alone forms homomeric channels displaying a very low amplitude current[11]. Coexpression of NR1 with NR2 subunit enhances the expression of functional channels[9,12]. Enhancement of NMDA receptor function has been shown to occur after chronic morphine exposure, which also appears rapidly during 4, 6, and 8 nM remifentanil infusion[6,13,14]. OIH can be prevented by NMDA receptor antagonist ketamine both in animals and humans. Therefore, NMDA receptors-mediated pain facilitation is an important potential mechanism of OIH[15].

Glycogen synthase kinase-3 (GSK-3) is a multifunctional serine/threonine protein kinase and ubiquitous in eukaryotes. In mammals, GSK-3 has two subtypes, GSK-3α and GSK-3β[16]. It plays a fundamental role in a wide variety of functions, including glycogen metabolism, cell differentiation and proliferation[17]. Considerable studies show that GSK-3β is a vital regulator in axon growth and neuronal polarity during development[18]. Recent studies have found that GSK-3β affects synaptic plasticity via regulating NMDA receptor’s trafficking, and GSK-3β inhibitors can restrain NMDA receptor expression in the postsynaptic membrane[19,20]. Parkitna et al[21] reported that GSK-3β inhibitors abolished development of morphine-induced hyperalgesia and tolerance in rats. Recently, we found that GSK-3β could regulate spinal cord NMDA receptor trafficking in a rat model of remifentanil-induced postoperative hyperalgesia[22]. However, the mechanism underlying remifentanil-induced hyperalgesia is still not well understood.

The aim of this study was to investigate whether GSK-3β inhibition could prevent remifentanil-induced hyperalgesia via regulating spinal NMDA receptor expression and function in vivo and in vitro.

## Materials and Methods

### Ethics Statements

All experimental procedures and protocols were approved by the Institutional Animal Care Committee of Tianjin Medical University and performed according to the “Policies on the Use of Animal and Humans in Neuroscience Research”. The protocol was approved by the Committee on the Ethics of Animal Experiments of Tianjin Medical University General Hospital, Tianjin, China (Permit Number: 2011-X6-18). All surgery was performed under Chloral Hydrate anesthesia, and all efforts were made to minimize suffering and to use the minimum number of animals necessary to obtain valid results.

### Animals

Experiments were performed on adult (weighing 240-260 g) and newborn (14-21day old) male Sprague-Dawley (SD) rats in vivo and in vitro, respectively. All animals were obtained from the Laboratory Animal Center of Academy of Military Medical Sciences of the Chinese People’s Liberation Army. Animals were housed in cages with a 12 h light-12 h dark cycle (lights on at 7:00 AM) at a constant room temperature of 22 ± 2 °C. The animals had access to food and water *ad libitum*. 

### Experimental Protocol

The rats were anesthetized with an intraperitoneal injection of 400 mg/kg chloral hydrate. Then the rats were placed in plastic tubular restrainers. A 24-gauge over-the-needle Teflon catheter was inserted into caudal vein and flushed with heparinized saline.

1 To investigate the lowest remifentanil (Ultiva®, USA) infusion rate and dosage which can induce hyperalgesia, forty adult rats were randomly divided into 5 groups (n = 8 in each group): saline group (Saline, 0.1 ml·kg^-1^·min^-1^, 60 min, iv) and 4 remifentanil groups (Rem, 0.25 μg·kg^-1^·min^-1^, 0.5 μg·kg^-1^·min^-1^, 1.0 μg·kg^-1^·min^-1^ or 2.0 μg·kg^-1^·min^-1^, 60 min, iv). The thermal and mechanical hyperalgesia was measured at baseline (-24 h) and 2 h, 6 h, 24 h, 48 h after remifentanil or saline infusion by paw withdrawal latency (PWL) and paw withdrawal thresholds (PWT), respectively.
2 Based on the above experiment, remifentanil infusion at a rate of 1.0 μg·kg^-1^·min^-1^ was selected to induce hyperalgesia. To investigate whether GSK-3β inhibition could prevent remifentanil-induced hyperalgesia via regulating spinal NMDA receptor expression in vivo, another 60 adult rats were randomly divided into 5 groups (n = 12 in each group): saline group (C group, 0.1 ml·kg^-1^·min^-1^, 60 min, iv), Glycine group (G group, 15 μg·kg^-1^·min^-1^, 60 min, iv; Glycine is an accessory in the pharmaceutical preparation of remifenanil), remifentanil group (R group, 1.0 μg·kg^-1^·min^-1^, 60 min, iv), remifentanil plus TDZD-8 (a GSK-3β inhibitor, Sigma, Canada) group (RT group, remifentanil 1.0 μg·kg^-1^·min^-1^ and TDZD-8 1.0 μg·kg^-1^, 60 min, iv) and TDZD-8 group (T group, normal saline 0.1 ml·kg^-1^·min^-1^ and TDZD-8 1.0 μg·kg^-1^, 60 min, iv). The thermal and mechanical hyperalgesia was evaluated by PWT and PWL at baseline (-24 h) and 2 h, 6 h, 24 h, 48 h after infusion. After the last behavioral test (48 h after infusion), spinal cord segments L_4_-L_5_ were harvested to evaluate NMDA receptors (NR1, NR2A and NR2B), phosphorylated GSK-3β and total GSK-3β expression by western blot.
3 To investigate whether GSK-3β inhibition could prevent remifentanil-induced hyperalgesia via regulating spinal NMDA receptor function in vitro, another 32 young SD rats were divided into 4 groups (n = 8 in each group) to run whole cell patch clamp recording test: Control group [C group, the spinal slices were only incubated with artificial cerebral spinal fluid (ACSF) for 60 min], Glycine group (G group, the spinal slices were incubated in ACSF with 0.24 μM glycine for 60 min), Remifentanil group (R group, the spinal slices were incubated in ACSF with 4 nM remifentanil for 60 min), Remifentanil plus TDZD-8 group (RT group, the spinal slices were incubated in ACSF with 4 nM remifentanil and 10 μM TDZD-8 for 60 min). After incubation, the NMDA receptor-mediated miniature excitatory postsynaptic current (mEPSC) was detected to evaluate the function of NMDA receptor.

### Behavioral Testing

All behavioral tests were performed by a person who was blind to the experimental groups. To evaluate mechanical hyperalgesia, PWT was determined by electronic Von Frey filaments (BSEVF3, Harward Apparatus Co., USA). Adult rats were placed individually in a cage (20 cm × 20 cm × 20 cm) with a wire mesh bottom (1 cm × 1cm). Von Frey filaments were applied vertically to the plantar side of right hind paw. Each trial was repeated five times at 15 min interval. A positive response was defined as complete lifting of the hind paw off the surface of the cage or flinching. A maximal cut-off value of 50 g was used to prevent tissue damage.

To evaluate thermal hyperalgesia, rats were placed into a clear plastic chamber on a hot plate (YLS-6B, Huaibei Zhenghua, Biological Instrument Equipment Co., Ltd., China). The hot plate is a round heated surface surrounded by plexiglass and maintained at 55 °C. The device is connected to a manually operated timer that records the amount of time the rat spends on the heated surface before showing signs of nociception (e.g. jumping, paw licks). Each trial was repeated five times at 15 min interval. A cut-off time of 40 s was used to avoid tissue damage to the hind paw.

### Western Blot

The rats were anesthetized with an intraperitoneal injection of 400 mg/kg chloral hydrate. The L_4_-L_5_ spinal cord segments were removed rapidly and stored in liquid nitrogen after finishing behavioral tests (48 h after infusion). To prepare a total lysate, the dorsal horn of the spinal cord was homogenized in ice-cold lysis buffer (50 mM Tris, pH 7.5, 150 mM NaCl, 2% Triton X-100, 100 g/ml phenylmethylsulfonyl fluoride, 1 g/ml aprotinin, and phosphatase inhibitors). The lysate was centrifuged at 12, 000 g for 30 min at 4 °C. A membrane compartment protein extraction kit (Biochain Institute, Inc., Hayward, CA) was used to extract the membrane fraction of the dorsal horn. The membrane and total protein were detected by Western blot with mouse anti-rat epidermal growth factor receptor (EGFR, 1:2,000; MBL, Naka-ku Nagoya, Japan) and monoclonal mouse anti-β-actin antibody (1:5,000; Sigma-Aldrich, USA), respectively. Samples (20 μg protein) were adjusted to a similar volume with loading buffer (10% sodium dodecyl sulfate, 20% glycerin, 125 mM Tris, 1 mM EDTA, 0.002% bromphenol blue, 10% β-mercaptoethanol), and the protein was denatured by heating at 95 °C for 5 min. Samples were separated on 10% SDS-PAGE, and transferred onto nitrocellulose membrane. The membranes were blocked with 5% nonfat milk in Tris-Tween buffer saline for 1 h (TBST; 50 mM Tris-HCl, 154 mM NaCl, and 0.05% Tween 20, pH 7.4), incubated overnight at 4 °C with polyclonal rabbit antibodies against rat NR1, NR2A, NR2B (all 1:300 dilution in 5% nonfat milk in TBST, Chemicon, USA) or rabbit anti-rat GSK-3β and phosphorylated (ser9) GSK-3β antibodies (all 1:1000 dilution in 5% nonfat milk in TBST, Cell Signaling Technology, USA), then incubated with horseradish peroxidase-conjugated goat anti-rabbit IgG antibodies (1:2, 000 in 5% nonfat milk in TBST, Jackson Immuno Research, USA) for 1 h. Membrane bound secondary antibodies were detected using Chemiluminescence plus reagent (Perkin Elmer Life and Analytical Sciences, USA) and visualized using a chemiluminescence imaging system (Syngene, Cambridge, UK). The Western blot analysis was repeated five times. The density of each specific band was measured using a computer-assisted imaging analysis system (Gene Tools Match software; Syngene, Cambridge, UK).

### Spinal Cord Slices Preparation and Whole-cell Patch-clamp Recording

The method used for obtaining rat spinal cord slices is described previously [23,24]. The rats were anesthetized with an intraperitoneal injection of 400 mg/kg chloral hydrate. The lumbosacral spinal cords (L_4_-L_5_) were separated by an anterior approach and sliced into transverse chips (350 μm) with a vibratome (VT1000S, Leica, Germany). Then, the slices were incubated in ACSF at room temperature (22 °C-25 °C), and aerated with 95% O_2_ and 5% CO_2_ at pH 7.4 for 60 min. The components of ASCF are (in mM): 126 NaCl, 3.5 KCl, 1.25 NaH_2_PO_4_, 26 NaHCO_3_, 2 MgCl_2_, 2 CaCl_2_, 10 D-glucose.

Spinal cord slices were individually transferred into a recording chamber which was continuously perfused with oxygenated ACSF and placed on an upright microscope equipped with infrared differential interference contrast optics (BX51W1, Olympus, Japan). The individual neurons can be identified through the television monitor connected to a low light sensitive CCD camera (710M, DVC, USA). Whole-cell patch-clamp recording was made from the dorsal horn neurons with microelectrodes. The vertical electrode puller (PIP5, HEKA, Germany) was used to produce borosilicate glass patch electrodes with tip openings of 1~2 μm and a series resistance of 3~5 MΩ. Electrodes were filled with an intracellular solution containing (in mM): 130 KCl, 10 HEPES, 0.5 CaCl_2_, 10 EGTA, 2 MgCl_2_, 2 Mg-ATP, and 0.3 Na-GTP, pH 7.3. To make sure that the mEPSC was specifically mediated by NMDA receptor, CNQX (20 μM), tetrodotoxin (TTX 10 μM) and bicuculline (BIM 20 μM) were added to the perfusion slot solution at the same time before recording. In previous trials, AP-5 (2 μM), a NMDA receptor antagonist, was added after NMDA receptor-induced mEPSC was detected and mEPSC would fade away a few minutes later. So, the mEPSC was mediated by NMDA receptors. To observe NMDA receptor-mediated synaptic responses, we used the ACSF with no Mg^2+^. All the recordings were made under room temperature (22 °C-25 °C). All responses were collected using an EPC 10 amplifier and Pulse 8.52 software (HEKA, Germany). Currents were filtered at 2.9 kHz with an eight-pole, low-pass Bessel filter and digitized at 10 kHz for later off-line analysis. Clampfit 9.0 (Axon Instruments, USA) was used to analyze mEPSC. The series resistance ranged from 10 to 30 MΩ after break-in and recordings or series resistance changed significantly were discarded. Recordings with seals <1 GΩ or resting membrane potentials greater than − 60 mV were excluded in the analysis. And the total charge transfer by all mEPSC was determined for each condition in a period of 5 min. Every trial was repeated eight times.

### Statistical Analysis

Values were expressed as mean ± SEM. Time course data for both the thermal and mechanical hyperalgesia were analyzed by two-way ANOVA with repeated measures to detect interactions between treatment and time. ANOVAs with statistically significant interactions between treatment and time (*P* < 0.05) were followed by *post hoc* comparisons using Bonferroni’s *t* test when appropriate. Western blot data was analyzed by one-way ANOVA followed by Tukey-Kramer *post-hoc* analysis. Cumulative probability of amplitudes and inter-event intervals of mEPSC in different groups were analyzed with Kolmogorov-Smirnov test. Statistical analysis was performed with GraphPad Prism 5.0 (GraphPad Software Inc, La Jolla, CA). *P* < 0.05 was considered statistically significant.

## Results

### Remifentanil-induced Mechanical and Thermal Hyperalgesia

**Figure 1:**
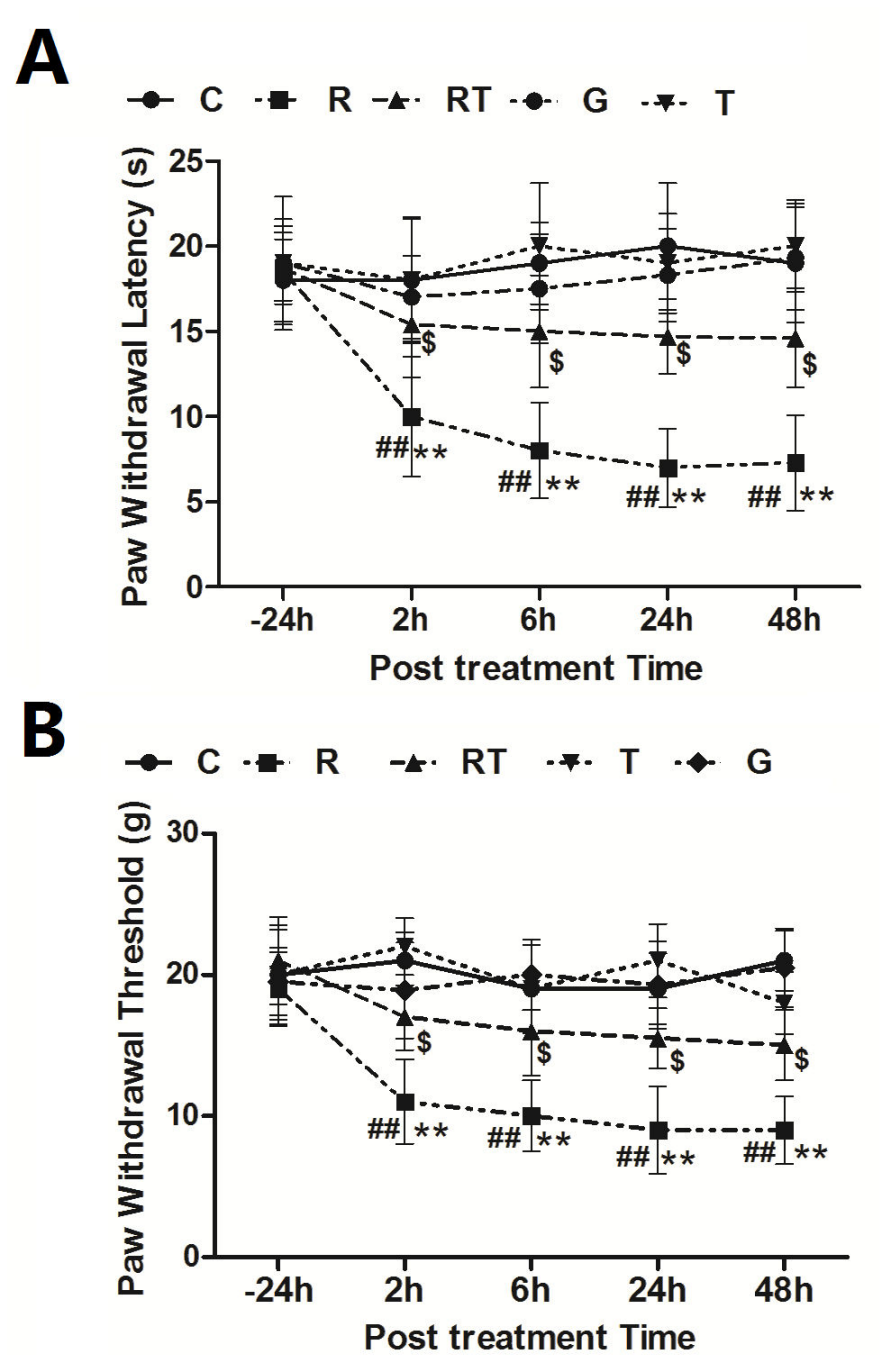
GSK-3β inhibition prevents remifentanil-induced thermal (A) and mechanical (B) hyperalgesia. Sixty adult rats were randomly divided into 5 groups (n = 12 in each group): saline group (C group, 0.1 ml·kg^-1^·min^-1^, 60 min, iv), Glycine group (G group, 15 μg·kg^-1^·min^-1^, 60 min, iv; Glycine is an accessory in the pharmaceutical preparation of remifenanil), remifentanil group (R group, 1.0 μg·kg^-1^·min^-1^, 60 min, iv), remifentanil plus TDZD-8 (a GSK-3β inhibitor, Sigma, Canada) group (RT group, remifentanil: 1.0 μg·kg^-1^·min^-1^ and TDZD-8: 1.0 μg·kg^-1^, 60 min, iv) and TDZD-8 group (T group, normal saline: 0.1 ml·kg^-1^·min^-1^ and TDZD-8: 1.0 μg·kg^-1^, 60 min, iv). Thermal latency to noxious heat and mechanical paw withdraw threshold were recorded at baseline (-24 h) and 2 h, 6 h, 24 h and 48 h after infusion. The antihyperalgesic effect of TDZD-8 was shown in remifentanil-induced hyperalgesia rats. Compaired with baseline (-24 h), ^##^ *P* < 0.01. Compaired with C group, * *P* < 0.05, ** *P* < 0.01. Compaired with R group, ^$^ *P* < 0.05.

 The lowest rate and dosage of remifentanil infusion which could induce hyperalgesia was determined by testing the mechanical and thermal hyperalgesia at baseline (- 24 h) and 2 h, 6 h, 24 h, 48 h after infusion. Compared with Saline group, remifentanil infusion at 1 and 2 μg·kg^-1^·min^-1^ for 60 min caused the significant decrease of PWT and PWL from 2 h to 48 h (*P* < 0.01 and *P* < 0.01, respectively, Figure S1A and B). No significant change of PWT and PWL was observed in remifentanil infusion at 0.25 and 0.5 μg·kg^-1^·min^-1^ (*P* > 0.05 and *P* > 0.05, Figure S1A and B). Glycine is an accessory in the pharmaceutical preparation of remifenanil and the ratio of glycine to remifentanil is 15 : 1. After glycine infusion (15 μg·kg^-1^·min^-1^, 60 min), there was no significant difference on PWL and PWT when compared with Saline group (C group) (*P* > 0.05 and *P* > 0.05, Figure 1A and B). Those results suggest that remifentanil infusion at a relative lower rate (1 μg·kg^-1^·min^-1^) can induce mechanical and thermal hyperalgesia. And glycine infusion at the rate of 15 μg·kg^-1^·min^-1^ had no effect on mechanical and thermal hyperalgesia.

### GSK-3β Inhibition Attenuates Remifentanil-induced Mechanical and Thermal Hyperalgesia

Based on the above experiment, remifentanil infusion at the rate of 1 μg·kg^-1^·min^-1^ was used in the following experiments. To investigate whether GSK-3β participates in remifentanil-induced hyperalgesia, a selective GSK-3β inhibitor TDZD-8 (1 μg·kg^-1^) was iv infused with remifentanil. We found that TDZD-8 significantly improved the changes of PWT and PWL in remifentanil-treated rats (*P* < 0.05, Figure 1A and B). Rats treated with remifentanil and TDZD-8 showed significant decrease on PWT and PWL when compared with saline group (*P* < 0.05, Figure 1A and B). GSK-3β inhibition alone has no direct effect on behavioral results when compared with saline group (*P* > 0.05, Figure 1A and B). Those results suggest that GSK-3β inhibition partially attenuated remifentanil-induced thermal and mechanical hyperalgesia. 

### GSK-3β Activity in Spinal Dorsal Horn is Increased after Remifentanil Infusion

**Figure 2:**
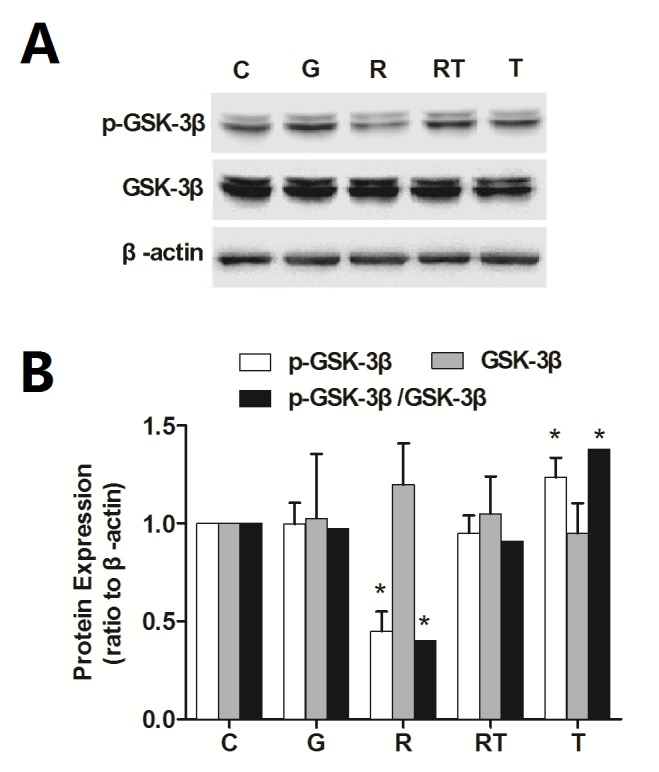
Remifentanil infusion increases the GSK-3β activity in spinal dorsal horn. The total GSK-3β and phosphorylated GSK-3β in spinal dorsal horn were tested by Western blot. β-actin was used as the internal standard (a). The band intensity of C group was assigned a value of 1. Remifentanil resulted in significant decreases of pGSK-3β (ser9) and p-GSK-3β (ser9)/GSK-3β ratio, but had no effect on the total protein level of GSK-3β (b). GSK-3β inhibitor TDZD-8 prevented the changes of pGSK-3β (ser9) and pGSK-3β (ser9)/GSK-3β ratio. n = 5 for each group. Compaired with C group, * *P* < 0.05, ANOVA.

The activity of GSK-3β is reflected by the ratio of pGSK-3β (Ser9) / GSK-3β. We therefore examined the protein expression of total lysate and Serine 9 phosphorylation of GSK-3β in spinal dorsal horn by western blot. As shown in Figure 2A and B, remifentanil infusion caused the significant decrease of pGSK-3β (Ser9) expression and pGSK-3β (Ser9) / GSK-3β ratio (*P* < 0.05, vs C group), but had no significant effect on the total protein level of GSK-3β (*P* = 0.66, vs C group). There is no significant difference in pGSK-3β (Ser9) and pGSK-3β (Ser9) / GSK-3β ratio between C and RT groups (*P* = 0.83 and *P* = 0.53, respectively). Together, these results show that remifentanil infusion can increase the GSK-3β activity in spinal dorsal horn by reducing the phosphorylation at serine 9 residue, which is prevented by TDZD-8.

### GSK-3β Regulates the Expression of NMDA Receptors in Spinal Dorsal Horn

**Figure 3:**
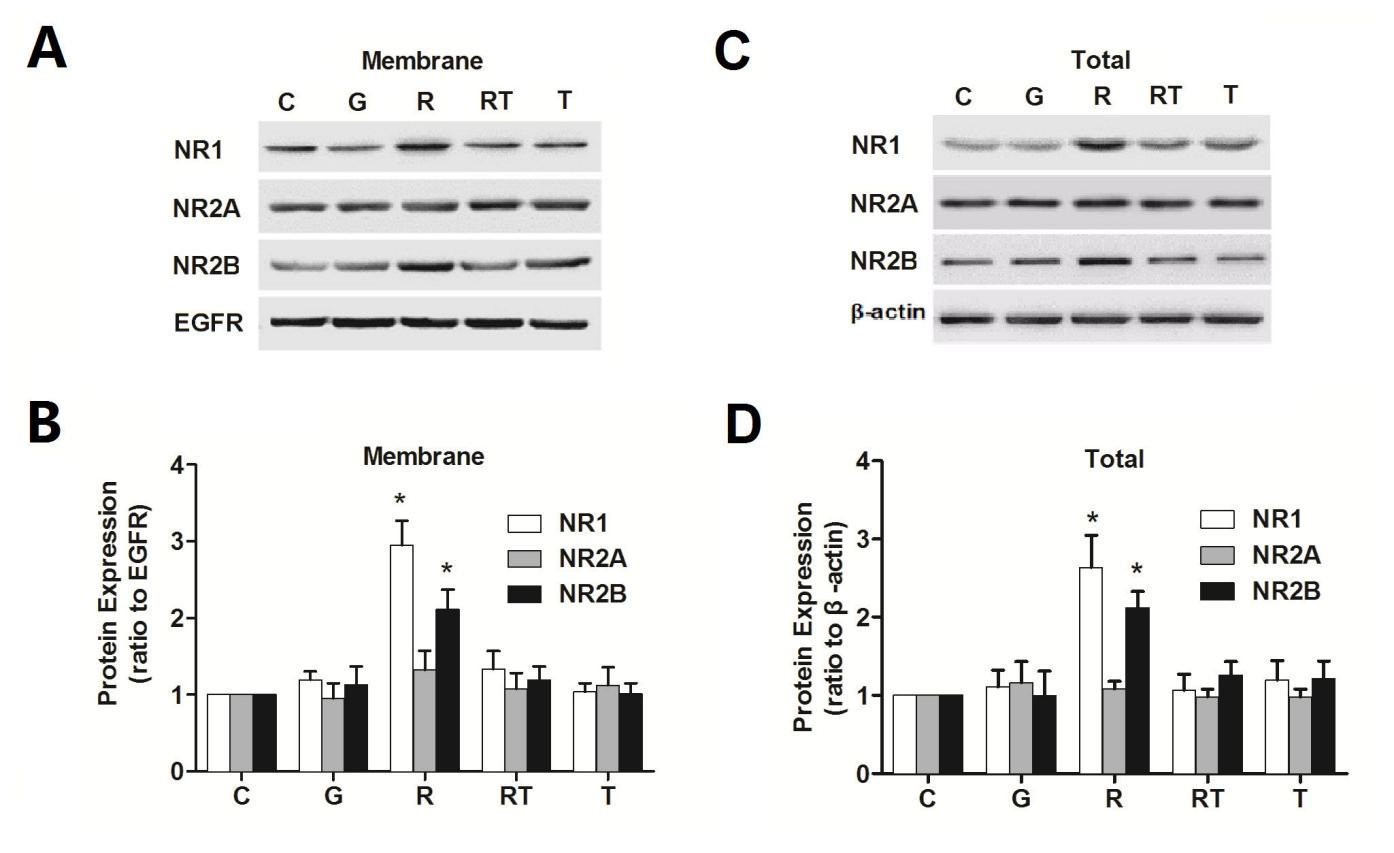
GSK-3β regulates the expression of NMDA receptors in spinal dorsal horn. Western blot for membrane NR1, NR2A and NR2B subunit was performed on rat spinal cord dorsal horn neuron (A). Epidermal growth factor receptor (EGFR) was used as the loading control. Pooled densitometric results for NR1, NR2A and NR2B, with the band intensity of C group assigned the value of 1. Remifentanil induced significant increases of both membrane NR1 and NR2B, but had no effect on membrane protein level of NR2A. GSK-3β inhibitor TDZD-8 prevented the changes of membrane NR1 and NR2B. n = 5 for each group, compared with C group, * *P* < 0.05, ANOVA (B). The expression of total NR1, NR2A and NR2B protein in spinal dorsal horn was tested by Western blot (C). β-actin was used as the loading control. Densitometry measurements from 5 groups were pooled and the band intensity of C group was assigned a value of 1. Remifentanil increased the total protein level of NR1 and NR2B, but had no effect on total protein level of NR2A. TDZD-8 prevented the changes of total protein expression of NR1 and NR2B (D). n = 5 for each group, compared with C group, * *P* < 0.05, ANOVA.

As shown in Figure 3A and B, the increased levels of membrane NR1 and NR2B subunits in spinal dorsal horn were seen in remifentanil-treated animals. TDZD-8 treatment (RT group) could prevent the increase of membrane NR1 and NR2B in remifentanil-treated animals (Figure 3A and B). Total protein levels of NR1 and NR2B were also increased after remifentanil infusion (R group, NR1, *P* < 0.05; NR2B, *P* < 0.05), which was also prevented by TDZD-8 treatment (RT group, Figure 3C and D). However, there is no significant difference in either membrane or total protein level of NR2A in all groups (Figure 3). In addition, glycine had no effect on the expression of NR1, NR2A and NR2B subunit (Figures 3). These results indicate that GSK-3β inhibition could prevent remifentanil-induced NR1 and NR2B expression and membrane trafficking in spinal dorsal horn.

### Remifentanil Increases NMDA Receptor Function in Spinal Dorsal Horn Neurons

**Figure 4:**
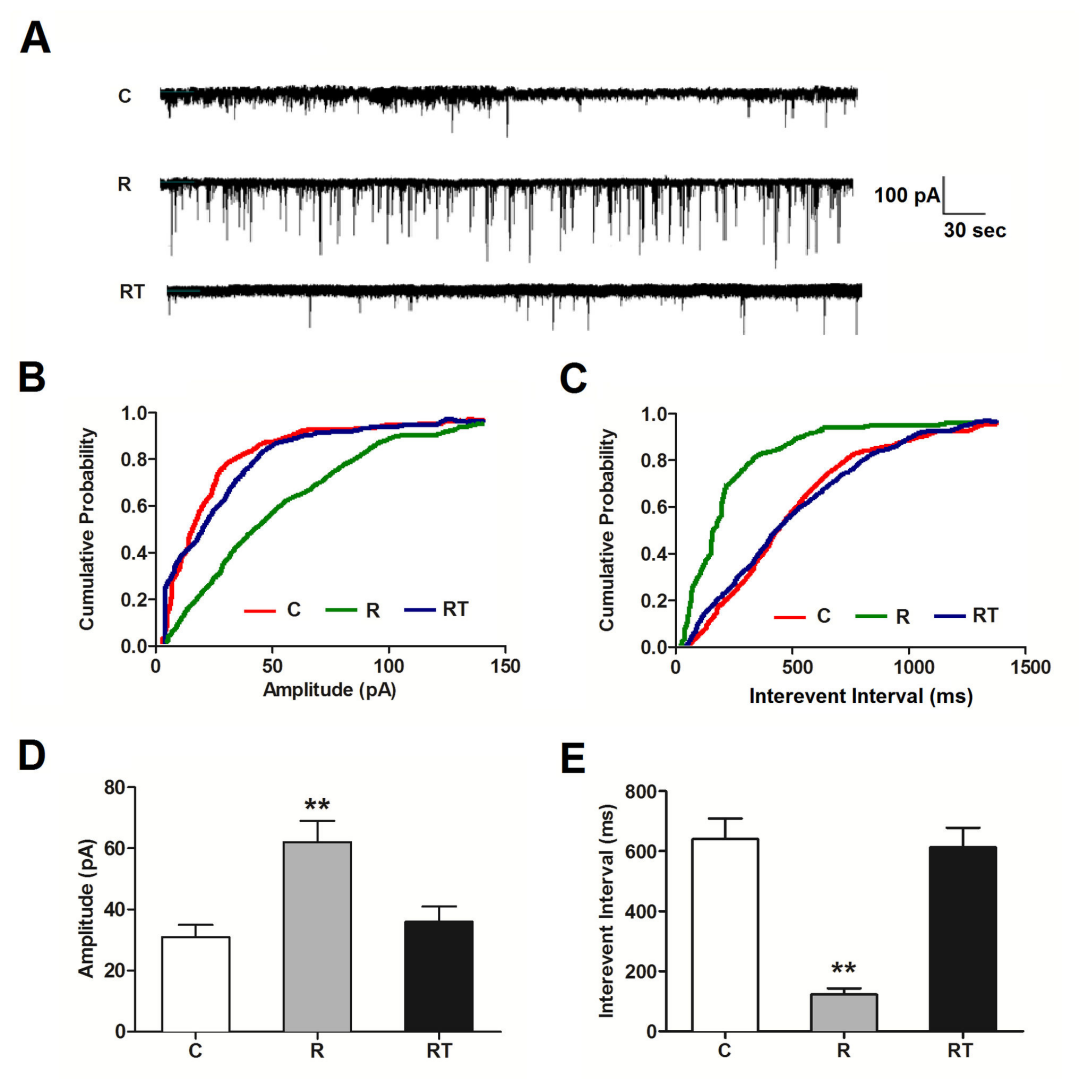
GSK-3β inhibition prevents the enhancement effect of remifentanil on NMDA receptor-mediated mEPSCs in dorsal horn neurons NMDA receptor-mediated mEPSCs in dorsal horn neurons were recorded at the holding potential of -70mV in the presence of TTX (10 μM), GABA receptor antagonist bicuculline (BIM, 20 μM) and AMPA receptor antagonist CNQX (20 μM). Representative traces of mEPSCs under control conditions (C group) and Remifentanil (R group) and Remifentainil+TDZD-8 treatment (RT group) were showed in A. *Scale* *bar*, 100 pA, 30 s. Cumulative probability plots of mEPSCs amplitude distribution showed significant shift after remifentanil treatment in spinal dorsal horn neurons (Kolmogorov-Smirnov test, *P* < 0.01), but no shift after Remifentainil+TDZD-8 treatment (Kolmogorov-Smirnov test, *P* = 0.881), (B). The distribution of cumulative interevent intervals (IEIs) for mEPSCs showed significant shift after remifentanil treatment in spinal dorsal horn neurons (Kolmogorov-Smirnov test, *P* < 0.01), however there is no shift after Remifentainil+TDZD-8 treatment (Kolmogorov-Smirnov test, *P* = 0.932), (C). Summary bar graph of mEPSCs amplitude in dorsal horn neurons treated with different treatments (D). n = 8 for each group, vs C group, **P < 0.01, ANOVA. Bar graph of mEPSCs IEIs in dorsal horn neurons treated with different treatments (E). n = 8 for each group. vs C group, **P < 0.01, ANOVA.

In order to make sure that 4 nM remifentanil has the ability to increase the response of NMDA receptor, whole-cell recordings were made to detect NMDA receptor-mediated mEPSC of dorsal horn neurons. 4 nM remifentanil corresponds to the levels achieved with clinical infusion rate of 0.1-0.15 μg·kg^-1^·min^-1^. Whole-cell patch-clamp recordings were made at a holding potential of − 70 mV. Representative traces of NMDA receptor-mediated mEPSC with or without remifentanil were shown in Figure 4A. Cumulative probability plots were presented in Figure 4B, which summarized the amplitudes of NMDA-mediated mEPSC recording in the Control and remifentanil-treated slices (n = 8, *P* < 0.01, Kolmogorov-Smirnov test). Remifentanil could increase the amplitudes of NMDA-mediated mEPSC (Figure 4D, n = 8, *P* < 0.01, ANOVA). The interevent intervals of NMDA receptor-mediated mEPSC cumulative probability plots were presented in Figure 4C (n = 8, *P* < 0.01, Kolmogorov-Smirnov test). Remifenanil decreased the NMDA-mediated mEPSC inter-event interval (Figure 4E, n = 8, *P* < 0.01, ANOVA). These results suggest that remifentanil can enhance NMDA receptor function in dorsal horn neurons.

In addition, we investigated the effect of glycine, an accessory of the pharmaceutical preparation of remifenanil, on NMDA receptor -mediated mEPSCs of spinal dorsal horn neurons. The ratio of glycine and remifentanil is 0.24 μM : 4nM in pharmaceutical preparation of remifenanil. Therefore, 0.24 μM glycine was applied to incubate the spinal cord slices in this study. The sample traces of NMDA-mediated mEPSCs of control and glycine group were shown in Figure S2A. The inter-event intervals and amplitude of NMDA-mediated mEPSCs cumulative probability plot were presented in Figure S2B and C. The data showed that 0.24 μM glycine had no effect on NMDA-mediated mEPSCs both in interevent interval (n = 6, *P* = 0.934, Kolmogorov-Smirnov test) and amplitude (n = 6, *P* = 0.893, Kolmogorov-Smirnov test).

### GSK-3β Inhibition Prevents the Remifentanil-induced Increase of NMDA Receptor Function in Spinal Dorsal Horn Neurons

To further verify the effect of GSK-3β on NMDA receptor-mediated mEPSC of dorsal horn neurons, we examined NMDA-mediated mEPSC under the presence of remifentanil (4 nM) and TDZD-8 (10 μM). Representative traces of NMDA receptor-mediated mEPSC were shown in Figure 4A. The cumulative probability plots of NMDA current amplitude and interevent intervals were presented in Figure 4B and C (n = 8, vs C group, *P* = 0.881, *P* = 0.932, Kolmogorov-Smirnov test). TDZD-8 prevented the increase of NMDA current amplitude and the decrease of NMDA receptor interevent intervals when compared with R group (Figure 4D and E, n = 8, *P* < 0.01, *P* < 0.01, ANOVA). These results suggest that GSK-3β inhibition can prevent the remifentanil-induced enhancement of NMDA receptor function in spinal dorsal horn neurons.

## Discussion

In the present study, we showed that intravenous infusion of remifentanil could induce a dose- and time-dependent thermal and mechanical hyperalgesia in adult rats, which was prevented by GSK-3β inhibition. Moreover, remifentanil infusion increased the GSK-3β activity in spinal dorsal horn by reducing the phosphorylation at serine 9 residue. Remifentanil infusion increased the membrane and total NR1 and NR2B expression in spinal dorsal horn, which were prevented by GSK-3β inhibition. In patch-clamp study, we found that remifentanil enhanced the amplitude and frequency of NMDA receptor-mediated mEPSC, which were also attenuated by inhibition of GSK-3β. These results suggest that GSK-3β inhibition can prevent the remifentanil-induced hyperalgesia via regulating NMDA receptor expression and function in spinal dorsal horn.

Intraoperative remifentanil infusion has been related to postoperative OIH. It has been reported that remifentanil stimulates different NMDA receptor subunit combination (NR1A/NR2A, NR1A/NR2B)[25]. Synaptic NMDA receptor number and subunit composition are not static, but changed dynamically in a cell-specific and synapse-specific manner during development and in response to neuronal activity or sensory experience[26]. Therefore, NMDA receptor expression and function are considered to be pivotal in the development of OIH. Emerging evidence suggest that remifentanil-induced hyperalgesia can be prevented by small-dose ketamine, implicating that NMDA receptors are involved in the mechanism of remifentanil-induced hyperalgesia[13,15]. However the clinical application of ketamine is limited due to side effects such as hallucinations, sedation, dizziness and somnolence[27]. Blockade of NMDA receptor function may have some side effects like ketamine did, such as hallucination and restlessness. The underlying mechanism of NMDA receptor in remifentanil-induced hyperalgesia should be studied more in-depth and detailed. 

In the current study, it was demonstrated that exposure to 0.24 μM glycine for 60 min had no effect on NMDA receptor expression or NMDA receptor-mediated mEPSC. But Guntz E, et al[28] shows that 3 mM glycine can increase the amplitude of NMDA receptors current. The NMDA receptor current recorded after application of remifentanil is related to the presence of glycine[27]. Perhaps, it is because the concentration of glycine is much higher or the different study processes were used. Anyway, the lower dose of glycine has no effect on NMDA receptor current and pain threshold in the present study. 

We used NMDA receptor-mediated mEPSC of rat’s dorsal horn neurons to distinguish presynaptic and postsynaptic mechanisms[23]. Central hypersensitivity was performed in dorsal horn neurons especially the most significant changes in NMDA receptor of dorsal horn neurons. According to the quantum theory of synaptic vesicular release, miniature postsynaptic currents are assumed to represent the spontaneous release of individual vesicles or quanta of neurotransmitter from the presynaptic membrane. Thus, the frequency of NMDA receptor-induced mEPSC is considered to be the presynaptic effects of experimental manipulation, whereas the amplitude of NMDA-mediated mEPSC is thought to reflect postsynaptic effects[23,29]. Therefore, the function of NMDA receptor and the synaptic transmission were evaluated by the amplitude and the frequency of NMDA receptor-mediated mEPSC[23].

GSK-3 is prominently expressed in CNS, especially in hippocampus, neocortex and spinal cord. GSK-3β isoform has been extensively studied, although the function of GSK-3α isoform has been somewhat neglected[30]. GSK-3β is not only a serine/threonine kinase involved in many cellular processes, but also a component of multi-protein NMDA receptor complex[31]. Peineau et al[32] investigated the role of 58 Ser/Thr protein kinases in LTD of CA1 pyramidal neurons and found evidence for only GSK-3 involved in NMDA receptor-dependent LTD, suggesting GSK-3 might contribute to the NMDA receptor trafficking and function. Recently, we found that GSK-3β could regulate spinal cord NMDA receptor trafficking in a rat model of remifentanil-induced postoperative hyperalgesia[22]. Our present study indicated that the GSK-3β activation after remifentanil exposure was due to serine 9 residue phosphorylation. GSK-3β inhibition leads to the suppression of NMDA receptor expression and function by both pre- and post-synaptic mechanism. 

 GSK-3β is a key regulator involved in mediating intracellular signaling, regulating neuronal plasticity, gene expression, and cell survival[33]. GSK-3β antagonist lithium can protect against NMDA receptor-mediated excitotoxicity neuronal death in both culture and rodent models[20,34]. This effect may be caused by two aspects. One is that excitotoxicity and neurodegeneration are mediated by GSK-3β through increasing glutamate release from the presynaptic membrane. Dynamin I phosphorylation by GSK-3 controls the activity-dependent bulk endocytosis of synaptic vesicles for glutamate reuptake in the synaptic cleft[35]. Neurotransmitter release is dependent on the efficient retrieval of synaptic vesicles from the nerve terminal plasma membrane[36]. So we consider that GSK-3β may increase the glutamate release to enhance the frequency of NMDA receptor-mediated mEPSC through increasing glutamate reuptake. The other aspect is that GSK-3β regulates NMDA receptor expression on the plasma membrane and NMDA receptor subunit composition. GSK-3β inhibitor induced the down-regulation of NMDA receptor current through increasing the Rab5-mediated and PSD-95-regulated NMDA receptor internalization via a clathrin/dynamin-dependent manner in cortical neurons[37]. It suggests that GSK-3β inhibitor-induced down-regulation of NMDA current is caused by the reduction of functional membrane NMDA receptors. NMDA receptors are heteromeric assemblies of NR1, NR2 and NR3 subunits which co-translationally assemble to form functional channels with different physiological and pharmacological properties and distinct patters of synaptic targeting[38,39]. After GSK-3β inhibitor treatment, dorsal horn neurons showed a markedly reduced level of surface NR1 and NR2B and a significantly increased level of internalized NR1 and NR2B without the changes of NR2A. The reduced expression of cell surface NR1 regulated by GSK-3β, consequently, reduced the amount of intracellular Ca^2+^[19]. These may explain how GSK-3β inhibitor decreases the NMDA receptor-mediated mEPSC. 

GSK-3β activity has been implicated in pain responses. In this study, TDZD-8, a selective GSK-3β inhibitor, attenuated remifentanil-induced thermal and mechanical hyperalgesia in rats. Morphine treatment increased GSK-3β activity in mice, and GSK-3β inhibitors prevent the development of tolerance[21,40]. Convincing evidence show that AR-A014418, a selective GSK-3β inhibitor, produces antihyperalgesia and antinociception in neuropathic pain model in mice[41,42]. The AR-A014418-dependent antinociceptive effects were induced by modulation of the glutamatergic system through metabotropic and ionotropic (NMDA) receptors[42].

In conclusion, the present study suggests that remifentanil-induced hyperalgesia may through GSK-3β activation to enhance NMDA receptor expression and current by both presynaptic and postsynaptic levels. The protective role of GSK-3β inhibitors against the central hypersensitivity in the spinal cord may provide a new drug target to treat remifentanil-induced hyperalgesia.

## Supporting Information

Figure S1 **Concentration- and time-dependence of remifentanil-induecd hyperalgesia.**
(TIF)

Figure S2 **Glycine has no effect on the frequency and amplitude of NMDA receptor-mediated mEPSCs in spinal dorsal horn neurons.**
(TIF)
