# Supplementary figures and images for: Inhibition of Glycogen Synthase Kinase-3β Prevents Remifentanil-Induced Hyperalgesia via Regulating the Expression and Function of Spinal N-Methyl-D-Aspartate Receptors In Vivo and Vitro
Source: PLoS One. 2013 Oct 16;8(10):e77790. doi: 10.1371/journal.pone.0077790 (PMC3797695; doi:10.1371/journal.pone.0077790)

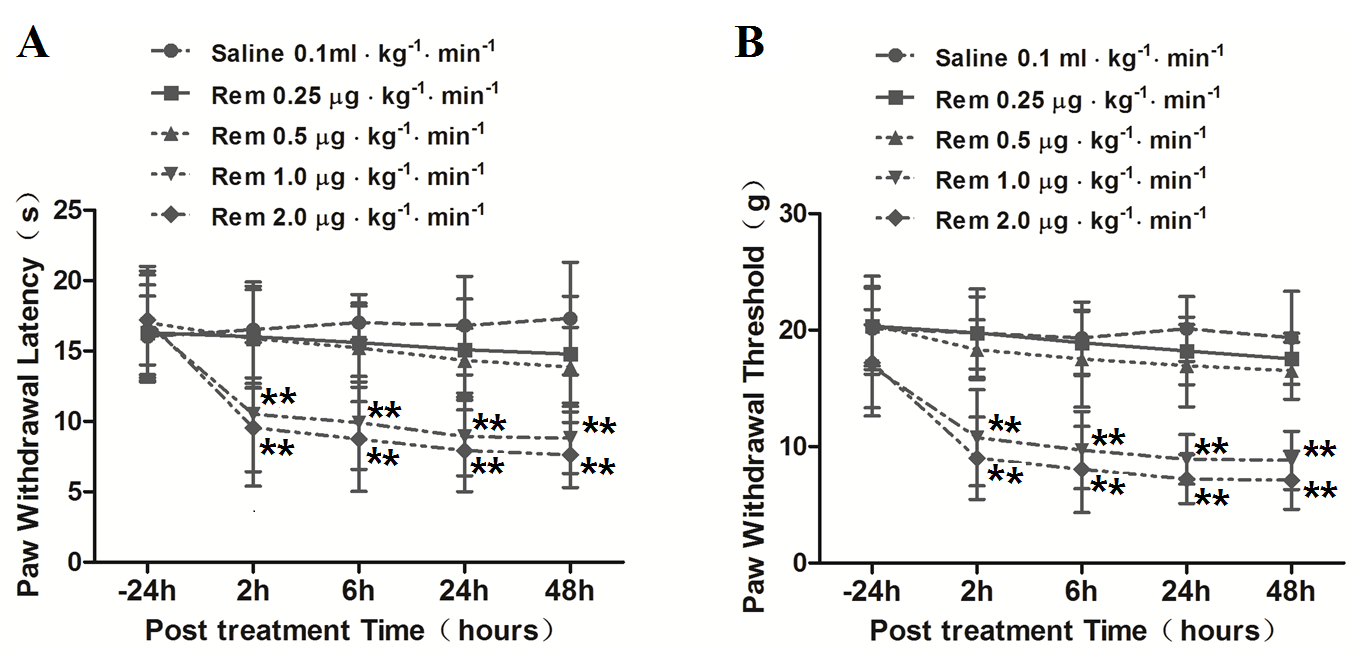

Supplement: Figure S1 — Concentration- and time-dependence of remifentanil-induecd hyperalgesia. (TIF) [file pone.0077790.s001.tif]

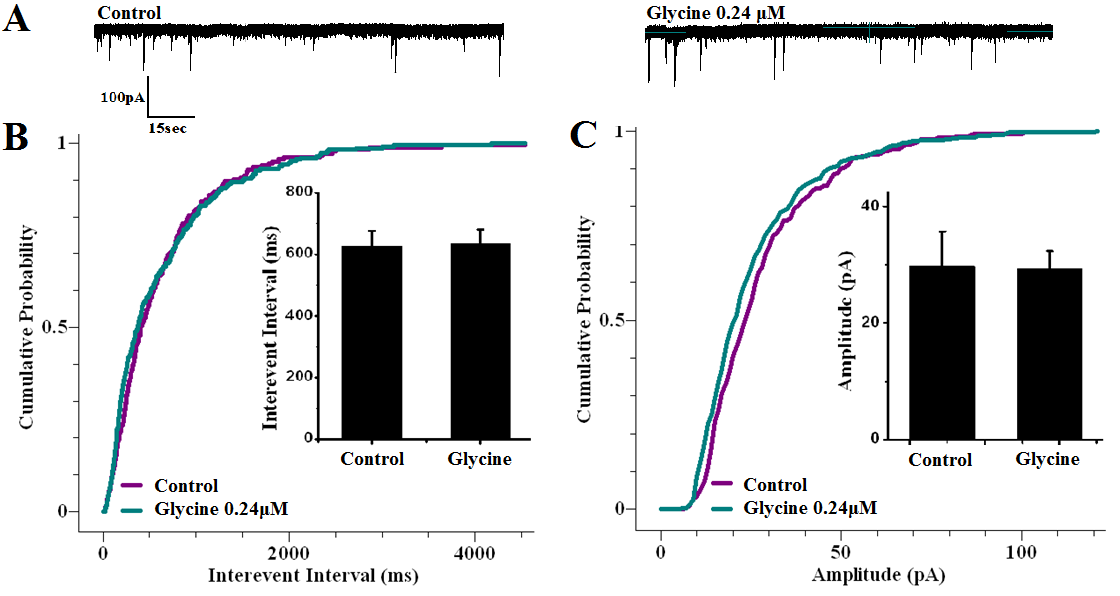

Supplement: Figure S2 — Glycine has no effect on the frequency and amplitude of NMDA receptor-mediated mEPSCs in spinal dorsal horn neurons. (TIF) [file pone.0077790.s002.tif]
